# Mucosal Microbiome Markers of Complete Pathologic Response to Neoadjuvant Therapy in Rectal Carcinoma

**DOI:** 10.1158/2767-9764.CRC-25-0036

**Published:** 2025-05-05

**Authors:** Ibrahim M. Abukhiran, Amr H. Masaadeh, James D. Byrne, Dustin E. Bosch

**Affiliations:** 1 Department of Pathology, Roy J. and Lucilla A. Carver College of Medicine, University of Iowa, Iowa City, Iowa.; 2 Department of Pathology, University of Pittsburgh Medical Center, Pittsburgh, Pennsylvania.; 3 Department of Radiation Oncology, Roy J. and Lucilla A. Carver College of Medicine, University of Iowa, Iowa City, Iowa.

## Abstract

The intestinal microbiome contributes to colorectal carcinogenesis, disease progression, and response to therapy. Pathologic complete response is the therapeutic goal of neoadjuvant chemoradiation in rectal carcinoma. Nonoperative management has become an accepted strategy, and markers of complete treatment response are needed. Intestinal commensal bacteria contribute to treatment response and radiation colitis, and microbiome-targeted therapies have shown promise in clinical trials. We investigated the relationship among mucosa-associated bacteria, neoadjuvant therapy response, and radiation colitis symptoms in 57 patients who received neoadjuvant regimens with no therapy, chemotherapy only, or chemoradiation. The design was a retrospective cohort study. Microbiome profiling was performed by 16S rDNA sequencing of formalin-fixed, paraffin-embedded tissue at the proximal margin of resection. Global β diversity differed according to neoadjuvant therapy modality and was associated with radiation colitis. Taxonomic differences were detectable at phylum and lower classification levels, and radiation-induced colitis was associated with enrichment of the *Bacillaceae* family. Taxonomic features, including reduced *Streptococcus*, *Lachnospiraceae*, and *Bacillaceae*, were enriched in complete histopathologic responders to neoadjuvant therapy. Taxon-based prediction of metabolic pathways identified enrichment of prokaryotic NAD^+^ biosynthesis and salvage pathways in complete responders. Mucosal microbiome responses to multimodal neoadjuvant therapy reflect symptomatic radiation colitis, histopathologic evidence of radiation injury, and pathologic treatment response. Posttreatment microbiome β diversity markers of complete pathologic response may be useful in decisions to manage rectal carcinoma nonoperatively.

**Significance:**

Posttreatment markers of the complete response of rectal carcinoma to neoadjuvant chemoradiation are needed to guide decisions about surgical resection. We found that mucosal microbiome β diversity, bacterial metabolic capacities, and specific taxonomic groups distinguished between complete and incomplete responders. The mucosal microbiome provides markers for complete pathologic response.

## Introduction

Dysbiosis of the intestinal microbiome contributes to colorectal carcinogenesis and disease progression (1). Known mechanisms of bacterial influence on colorectal cancer (CRC) initiation and progression include promotion of host cell mutagenesis through the production of genotoxins, promotion of oncogenic host signaling, and modulation of host antitumor immunity (2). Mucosal microbiomes likely differ by anatomic location (e.g., right- and left-sided colon cancers), suggesting that some bacterial influences on carcinogenesis and disease progression may be site specific (3). Components of the intestinal and primary tumor microbiomes are also found at distant metastatic sites (4). One of the most extensively studied pathobionts, *Fusobacterium nucleatum*, adheres to host enterocytes through Fap2 and stimulates Wnt signaling through FadA interactions with E-cadherin to promote carcinogenesis (5, 6). *F. nucleatum* colonizes 10% to 20% of CRC with predilection for right-sided location (7) and distant metastases (4).

Neoadjuvant radiation and/or chemotherapy are central components of rectal carcinoma management (8). Pathologic complete response, that is, no residual carcinoma identified at resection, currently occurs in 8% to 28% of patients with rectal carcinoma treated with neoadjuvant chemoradiation (9, 10). Pathologic complete response is associated with survival benefits, and clinical complete response enables consideration of possible omission of immediate posttreatment surgery (so-called “watch-and-wait”; ref. 11). As consideration of nonoperative management strategies occurs with increasing frequency, there is a need for improved posttreatment indicators of complete response (12). Response to chemotherapy and radiation is related to intestinal commensal bacteria (13). For example, nucleotide biosynthesis in *Phocaeicola vulgatus* is associated with resistance to neoadjuvant chemoradiotherapy, and gavage with *P. vulgatus* protects cancer cells from radiation in mouse xenograft models (14). Pretreatment intestinal microbiome composition may be useful in predicting the response to chemoradiation (15, 16).

Although pretreatment fecal microbiome studies provide valuable information about bacteria that may confer sensitivity or resistance to neoadjuvant therapy, current standard-of-care decisions to treat are dominated by clinical stage assessment (17). After completion of standard neoadjuvant chemoradiation, decisions to “watch-and-wait” or proceed with relatively morbid rectal surgery currently pose a greater challenge because of uncertainties with regard to the risk of recurrence and imperfect imaging and endoscopic indicators of complete response. Ultimately, the goal is to identify patients with no residual carcinoma (complete pathologic response) without resecting the treated rectum. Thus, posttreatment microbiome markers of complete response to neoadjuvant therapy may be useful in guiding management.

Intestinal inflammation (e.g., colitis or mucositis) is a common complication of both chemotherapy and radiotherapy (18). Approximately 25% of patients with advanced colorectal cancer experience high-grade diarrhea during treatment with irinotecan and oxaliplatin (19). Discontinuation of therapy due to intestinal inflammation and associated symptoms has deleterious effects on clinical outcomes (19). In addition to the acute symptoms of diarrhea, bleeding, and abdominal pain, radiotherapy carries the risk of long-term complications, including chronic diarrhea and intestinal obstruction (i.e., chronic radiation enteritis).

Several lines of evidence suggest that intestinal commensal bacteria and their interactions with the host contribute to the development of radiation enteritis and may represent a therapeutic target. Fecal microbiome α diversity decreases in patients treated with radiotherapy, particularly in those with diarrhea (20, 21). The intestinal microbiome mediates radiosensitivity in rodent models of total body irradiation. For instance, antibiotic therapy and fecal microbiota transplantation confer prolonged survival (22, 23). Pretreatment microbiome features may also be predictive of treatment-related symptoms. In patients with rectal carcinoma, pretreatment fecal microbiota differences are associated with co-occurring symptoms of depression, sleep disturbance, and fatigue (24). Specifically, the enrichment of some *Pseudomonadota* and *Turicibacter* taxa and the depletion of *Lachnospiraceae* were associated with the development of psychoneurologic symptoms.

Recent clinical trial evidence suggests efficacy of microbiome-targeted therapies for the treatment of radiation-induced colitis (25). Administration of VSL#3, a probiotic with four lactobacilli (*Lactobacillus casei*, *Lactobacillus plantarum*, *Lactobacillus acidophilus*, and *Lactobacillus delbrueckii* subsp. *bulgaricus*), three bifidobacteria (*Bifidobacterium longum*, *Bifidobacterium breve*, and *Bifidobacterium infantis*), and *Streptococcus salivarius* subsp. *thermophilus*, to patients undergoing adjuvant radiotherapy for colorectal or cervical cancer resulted in less radiation-related diarrhea and prolonged time to antidiarrheal therapy in a double-blind placebo-controlled trial (26). Several other probiotics and compounds derived from commensal bacteria have also shown effective protection against radiation-induced injury in a range of rodent models, e.g., *Lachnospiraceae* probiotics and short-chain fatty acids (SCFA; ref. 27). Small case series suggested that fecal microbiota transplantation may be beneficial in patients with chronic radiation enteritis (28). Antibiotic therapy may also improve rectal bleeding related to radiation (29, 30). However, probiotics and other microbiome-targeted therapies have not yet been included as the standard of care for radiation oncology patients.

Fecal and mucosa-associated microbiomes differ substantially. The nonuniform distribution of bacterial species relative to intestinal crypts and the mucus layer has been demonstrated in several studies (31–34). Bacterial stratification is mediated in part by microenvironmental conditions, such as oxygen gradients, physical barriers of the mucus layer, and host factors, such as mucosal immunity and secreted antibacterial peptides (31). Mucosal inflammation in radiation and chemotherapy injury is expected to disrupt the mucus barrier and alter mucosa-associated bacterial communities, as observed in other inflammatory colitides (35). Tissue-based microbiome profiling also allows the description of spatial patterns in bacterial communities and direct correlation with histologic features, such as vascular and epithelial changes associated with radiation injury and pathologic response of neoplasm to neoadjuvant therapy.

We hypothesized that posttreatment mucosal microbiome features may correlate with radiation colitis and treatment response. We designed a retrospective study of mucosa-associated commensal bacteria in rectal carcinoma resection specimens and compared patients receiving no neoadjuvant therapy, chemotherapy alone, or chemoradiotherapy. Patients receiving radiation were further stratified according to symptoms of radiation enteritis that required therapeutic intervention. Microbiome features were also compared with histopathologic features, including a radiation injury score (36) and pathologic treatment effect scores.

## Materials and Methods

### Ethics approval and consent to participate

All human subject research was performed in concordance with the Declaration of Helsinki. The study protocol was reviewed and approved by the University of Iowa Institutional Review Board (protocol 202110107). The requirement for written informed consent was waived by the institutional review board. No minors were included in the study protocol.

### Study design and case selection

**Figure 1:**
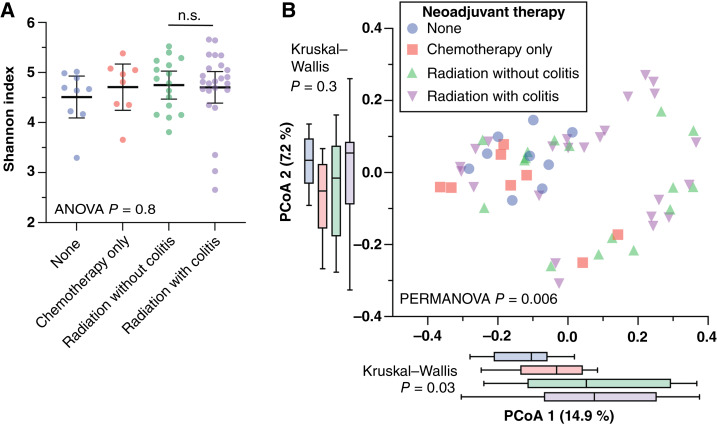
Mucosal microbiome β diversity is altered by neoadjuvant therapy and reflects colitis symptoms. Mucosa-associated bacteria were profiled using 16S rDNA sequencing from FFPE. **A,** α diversity did not differ among the treatment groups by several indices including Faith PD. **B,** Significant β diversity differences were detected with the PERMANOVA test, although clear separation of groups is not easily visualized by unweighted UniFrac principal component analysis (PCoA). n.s., not significant.

Rectal carcinoma resection pathology cases were retrospectively identified using a University of Iowa Health Care pathology database, which includes resections spanning from 2014 to 2023. Of the 100 cases examined for eligibility, 86 were confirmed to be eligible after exclusion of two subjects with inflammatory bowel disease, four with nonrectal or uncertain sigmoid versus rectal tumors, and eight referral cases with biospecimens not at Iowa. Twenty-nine of these cases were excluded for missing data upon health record and pathology review: five with slides and/or tissue blocks not available, five who received radiation that was less than 50.4 Gy/28 fractions (e.g., short-course radiation), six whose radiation dose was unknown, and 13 whose radiotherapy was managed at another institution. Fifty-seven were included in the study, all completing follow-up and analyzed. Subjects were grouped according to neoadjuvant therapy: 9 without neoadjuvant therapy (“none” group), 8 with neoadjuvant chemotherapy only, and 40 with neoadjuvant chemoradiotherapy. The neoadjuvant chemoradiotherapy group included 14 complete pathologic responders and 26 incomplete responders. Patients in the neoadjuvant chemoradiation group were selected and assigned as having “radiation with colitis” (24 patients) if the clinic notes of radiation oncologist visits described severe associated symptoms and loperamide was prescribed. The clinic visits in the “radiation without colitis” group of 16 patients lacked documented severe symptoms or the need for colitis symptom–directed therapy. The Common Terminology Criteria for Adverse Events were not used because scores were not routinely documented in clinical notes at our institution. The minimum cohort size was determined based on power calculation of ANOVA to test for α diversity differences across four groups (the test in Fig. 1A). Based on Shannon index means and variance from another of our studies with a similar design and an effect size of 0.5 U, four groups with ∼12 samples each were needed for α 0.05 and power 0.9.

Follow-up consisted of pathologic outcomes of treatment response and radiation injury histopathology score, obtained by slide and pathology report review. As all subjects chosen had complete data and no prospective follow-up was needed, there was no loss to follow-up. Table 1 shows the basic demographics, stage, and radiotherapy details. Chemotherapy included 5-fluorouracil (5-FU) or capecitabine in all patients, panitumumab in 5 patients, and bevacizumab in 2 patients. All resected specimens were collected in years 2015 to 2021. Most irradiated patients were treated by two radiation oncologists at the study institution, and six received radiotherapy at an outside institution. Twelve patients in the radiation colitis group and 4 in the no colitis group received chemotherapy followed by chemoradiation. All other patients received chemoradiation only.

**Table 1:** Study group demographics, stage, and oncologic treatment exposures

Variable	None	Chemotherapy only	Radiation without colitis	Radiation with colitis	Test	*P*
Total subjects, *n*	9	8	16	24	NA	NA
Sex, *n*					χ^2^	0.5
Male	4	2	9	13		
Female	5	6	7	11		
Age, median year (IQR)	53 (42–63)	47 (42–52)	64 (55–72)	60 (57–67)	Kruskal–Wallis	0.007
Pathologic stage group, *n*					χ^2^	0.7
1	4	1	5	11		
2	1	3	7	6		
3	3	4	4	7		
4	1	0	0	0		
Radiotherapy						
Interval to resection, median days (IQR)	NA	NA	75 (63–133)	108 (74–217)	Mann–Whitney	0.2
50.4 Gy/28, *n* (%)	NA	NA	16 (100)	24 (100)	NA	NA
Interval from colitis symptoms to resection, median days (IQR)	NA	NA	NA	105 (88–136)	NA	NA
Chemotherapy, *n*					χ^2^	0.01
5-FU based	NA	8	15	24		
Capecitabine	NA	1	11	21		
Oxaliplatin	NA	8	11	21		
Panitumumab	NA	5	0	0		
Bevacizumab	NA	2	0	0		
Microsatellite status, *n*					χ^2^	0.8
Instability (MSI)	1	0	0	0		
Stable (MSS)	8	8	16	24		
Postresection follow-up						
Follow-up interval, median months (IQR)	14 (8–28)	12 (7–19)	40 (30–43)	34 (30–43)	Kruskal–Wallis	0.02
Recurrence, *n*	1	0	1	1	χ^2^	1
Distant metastasis, *n*	1	1	2	1	χ^2^	0.8

Abbreviations: Gy, Gray; MSI, microsatellite instability; MSS, microsatellite stable; NA, not applicable.

### Histopathology, radiation injury, and treatment effect scoring

Slides were reviewed for all cases, and a radiation injury score was assigned according to previously published criteria (36). Briefly, scores were assigned from the sum of key histopathologic changes, each contributing subscore values of 0 to 3. As previously described, the assessed features included thickening of the serosa, mucosal ulceration, epithelial atypia, vascular sclerosis, intestinal wall fibrosis, and lymphatic congestion. All cases included in the study were reviewed and scored by two gastrointestinal pathologists and a pathology resident blinded to the treatment history. The plotted histopathology scores represent the mean of three independent observers. The treatment effect score was assessed according to the current guidelines of the College of American Pathology (37). Briefly, the scores were 0 for no viable cancer cells, 1 for single cells or rare small groups of cancer cells, 2 for residual cancer with evident tumor regression, and 3 for extensive residual cancer with no evident tumor regression.

### 16S rDNA PCR and sequencing

Microbiome profiling was performed on the proximal margins of resected specimens. Because all included specimens were low anterior resections or abdominoperineal resections, all margin sampling was located in the sigmoid colon. Nucleic acids were extracted from formalin-fixed, paraffin-embedded (FFPE) tissues using the QIAamp DNA FFPE Advanced kit (Qiagen) with uracil-N-glycosylase to mitigate cytosine deamination. Nonneoplastic tissue from the proximal margins of the rectal resections was selected, and the mucosal layer was dissected. 16S rDNA amplification, library generation, and sequencing were performed as previously described (38). Briefly, the V3 to V4 region of the 16S rRNA gene was amplified using primers 5′- TCGTCGGCAGCGTCAGATGTGTATAAGAGACAGCCTACGGGNGGCWGCAG-3′ and 5′-GTCTCGTGGGCTCGGAGATGTGTATAAGAGACAGGACTACHVGGGTATCTAATCC-3′. Indexing and library construction were performed using a second round of PCR. Multiplexed samples were sequenced by paired sequencing on a MiSeq instrument (Illumina, RRID: SCR_016379).

### Microbiome data analysis

We adapted a previously published protocol (38), designed for fecal metagenomic sequencing of the V3 to V4 variable regions of the 16S rRNA gene, to profile the mucosa-associated rectal microbiome of resection specimens from patients with rectal carcinoma. To assess mucosal microbiome differences away from the neoplasm, we microdissected the mucosa from the nonneoplastic large intestine at the proximal resection margin. The data were processed using QIIME 2 (RRID: SCR_021258; ref. 39). Sequence quality profiles were demultiplexed, and quality profiles were visualized using demux and summarize functions. The DADA2 pipeline (RRID: SCR_023519; ref. 40) was used for sequence quality control and feature table generation. Phylogenetic trees were generated using the QIIME 2 phylogeny function. α and β diversity metrics with group significance statistics were calculated using the q2-diversity plugin in QIIME 2. Taxonomic units were classified using a classifier trained on the Greengenes database (gg_12_8; RRID: SCR_002830; ref. 41). Differential abundance testing by linear discriminant analysis (LDA) was performed with LEfSe (RRID: SCR_014609) using a cutoff LDA score of >2.0 (42). Analysis outputs were visualized using QIIME 2 View (view.qiime2.org) and GraphPad Prism 9 (RRID: SCR_002798). Metabolic pathways were predicted based on taxonomy and relative abundance using PICRUSt2 (RRID: SCR_022647; ref. 43) and relative enrichment was detected using LDA.

### Exclusion of likely contaminant-derived sequences

Metagenomic sequencing of clinical FFPE colon tissue presents several challenges, including varying degrees of DNA damage and crosslinking related to formalin fixation, loss of bacteria and their DNA during tissue processing, nonspecific amplification of damaged host DNA, and introduction of contaminating bacterial DNA during specimen handling and tissue processing. To address this possibility, we applied two “decontamination” approaches. First, taxonomic groups that were overrepresented in 16S rDNA sequencing data from an independent set of 10 FFPE samples compared with paired fresh frozen colonic mucosa tissue from the same subjects were excluded as likely contaminants from routine FFPE tissue processing at our institution. The excluded taxonomic groups are listed (Supplementary Table S1), and the predominant group consisted of bacterial sequences that were not classifiable beyond the kingdom level (∼13% of all reads). In addition, we employed a previously published statistical approach for contaminant identification (R package decontam; ref. 44). There was a high concordance of likely contaminant identification by decontam and our frozen tissue/FFPE comparison (12 of 13 taxonomic groups, Supplementary Table S1). Analysis with decontam flagged 377 of 17,272 taxonomic units across all rectal carcinoma specimens as likely contaminants. Ultimately, ∼25% of the sequences were excluded as possible contaminants, dominated by kingdom bacteria sequences, not further classifiable (13%). After quality filtering and decontamination, there was a median of 51,488 reads per sample (IQR 18,506–117,645), comparable sequencing depth with a previous study of colon FFPE samples (45).

### Statistical analysis

α diversity was assessed using several metrics calculated using QIIME 2, including the Shannon index, Simpson index, Faith phylogenetic diversity (PD), and Chao1 index. Several metrics were employed because of their distinct sensitivities to microbiome alterations (46). The α diversity plots are representative of these multiple analyses, which showed consistent results in the statistical tests for our study. Statistical tests for α diversity were nonparametric, either Kruskal–Wallis or Mann–Whitney U tests depending on the number of groups compared, and performed using Prism (GraphPad). For β diversity analysis, a single metric of unweighted UniFrac distance was chosen prior to analysis and calculated using QIIME 2. β diversity was visualized using unweighted UniFrac distance metrics and principal component analysis, which included all the detected operational taxonomic units. Statistical testing for global differences across groups was performed using PERMANOVA in QIIME 2 (39). Phylum-level diversity was correlated with the study group using Kruskal–Wallis tests for each phylum’s relative abundance in Prism. The mean histology scores and relative abundances of individual taxa were compared across all study groups using the Kruskal–Wallis test in Prism. For LDA, a cutoff score of 2.0 was selected prior to analysis according to the default settings of LEfSe (42). Only taxa with an LDA score >2.0 in the LEfSe analysis were selected for representation in relative abundance comparisons.

### Data availability

The dataset supporting the conclusions of this article is available in the NCBI Sequence Read Archive repository under BioProject PRJNA1029660. The datasets used are available from the corresponding author upon reasonable request.

## Results

### Mucosal microbiome β diversity reflects neoadjuvant therapy modality and colitis symptoms

**Figure 2:**
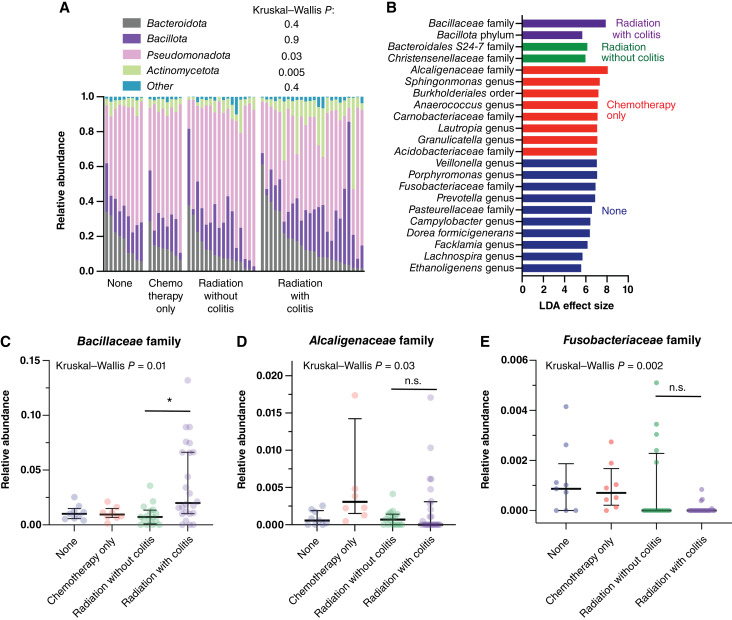
Taxa differ by neoadjuvant treatment modality and radiation colitis. **A,** Phylum-level abundances were related to the treatment group as detected with Kruskal–Wallis tests. **B,** Specific taxa were identified as differentially abundant across all treatment groups with LEfSe (42), using a default LDA effect score cutoff of 3.0. **C–E,** Relative abundances of taxa with high LDA effect size by treatment group are plotted. Relative abundances of *Bacillaceae* were higher in patients with radiation colitis, reaching ∼10% in several samples. Horizontal lines and error bars represent median and IQR. Asterisk represents a Mann–Whitney test *P* < 0.01. n.s., not significant.

We assessed the mucosa-associated microbiome of 57 patients with rectal carcinoma. Microbiome α diversity, measured using several indices, including Faith PD, Chao1, and Fisher indices (Shannon index shown in Fig. 1A, others in Supplementary Fig. S1), did not differ significantly among rectal carcinoma subjects across the study groups. However, a statistically significant β diversity difference was observed, as indicated by the UniFrac distance comparisons (PERMANOVA *P* = 0.006, Fig. 1B). At the phylum level, significant relative abundance differences among study groups were detected using Kruskal–Wallis tests (*P* = 0.03 and 0.005, Fig. 2A). Samples from patients with radiation colitis showed relative enrichment of *Actinomycetota* and depletion of *Pseudomonadota* compared with other subjects with neoadjuvant therapy. LDA [LEfSe (42)] comparing all four study groups (Fig. 2B) identified the *Bacillaceae* family as specifically enriched in the subset of irradiated subjects with colitis (Fig. 2C), reaching a relative abundance of ∼10% in several radiation colitis subjects. *Alcaligenaceae* were most abundant in the chemotherapy-only group (Fig. 2D). Although *Fusobacterium* spp. are most frequently associated with right-sided colorectal cancer (47), we observed a low abundance of *Fusobacteriaceae* in rectal carcinoma specimens, which decreased with radiation (Fig. 2E).

### Radiation injury severity score correlates with mucosal microbiome α diversity

**Figure 3:**
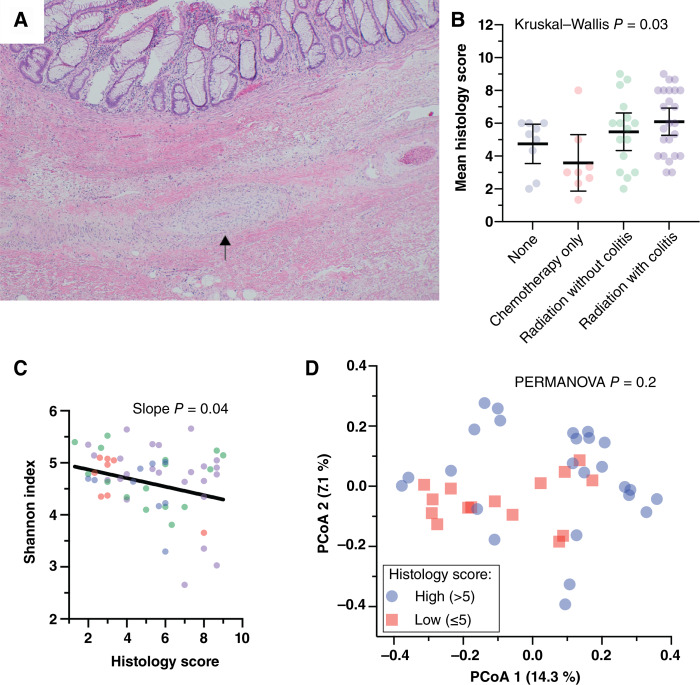
Mucosal microbiome α diversity is decreased with high radiation injury histology score. Radiation injury scoring was performed by three independent observers according to a previously published scheme (36). **A,** Representative hematoxylin and eosin–stained slide shows submucosal fibrosis and thickened blood vessels (arrows) in a rectum specimen following neoadjuvant radiation. **B,** Modest correlation with treatment modality was observed. **C,** α diversity was significantly lower in specimens with high radiation injury score. A *P* value represents a test of linear slope different from zero. Coloring is by group as in **A**. **D,** β diversity, measured with unweighted UniFrac distance, was not related to radiation injury histology score, which was arbitrarily divided near the median at score 5 (PERMANOVA test *P* = 0.2). PCoA, principal component analysis.

One possible explanation for the β diversity differences in patients with radiation colitis is that more severe rectal injury alters the mucosal microenvironment. To assess the severity of radiation injury, slides from rectal resection specimens for each subject were reviewed in a blinded fashion by three observers and scored using a previously described radiation injury severity score (Fig. 3A; see “Materials and Methods” for a description of the scoring components; ref. 36). The mean radiation injury severity scores differed between the groups and were higher in the chemoradiation group than in the chemotherapy alone group (Fig. 3B). However, there was high within-group variability. Radiation injury score components, such as epithelial atypia, are related to the timing and intensity of radiation exposure and can also be seen in response to chemotherapy (48). Lower α diversity was observed in cases with a high radiation injury severity score (Fig. 3C). Arbitrary division into high- and low-score radiation injury severity (score 5 chosen as near-midpoint) revealed no significant differences in β diversity (Fig. 3D). We conclude that one or more of the histologic parameters indicating mucosal injury are related to reduced α diversity of the mucosal microbiome.

### Mucosal β diversity is strongly related to the treatment effect score

**Figure 4:**
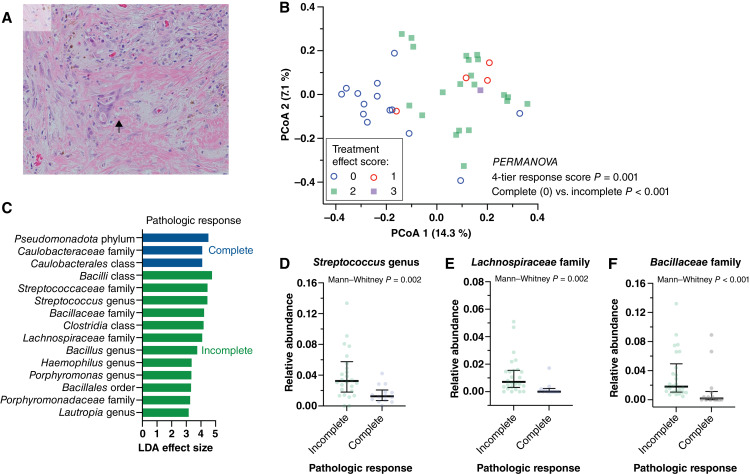
Microbiome β diversity distinguishes complete and incomplete pathologic neoadjuvant treatment response. Only the 40 patients treated with neoadjuvant chemoradiation were included in this analysis. **A,** Representative hematoxylin and eosin–stained slide from a rectum resection after neoadjuvant chemoradiation shows treatment effect with fibrosis and hemosiderin pigment, and rare small groups of residual carcinoma cells corresponding to a treatment response score of 1 (arrow). **B,** Unweighted UniFrac distances were significantly different comparing specimens with complete pathologic response to neoadjuvant treatment (treatment effect score of 0) with those with residual carcinoma (PERMANOVA *P* ≤ 0.001). PCoA, principal component analysis. **C,** LDA identified several taxa differentiating complete from incomplete pathologic response, including *Streptococcus*, *Lachnospiraceae*, and *Bacillaceae* (**D**–**F**).

The neoadjuvant treatment effect score is a histologic marker of rectal carcinoma response to radiation and chemotherapy, indicating the amount of remaining viable neoplasm on a 4-tiered scale from no residual carcinoma (score 0) to no detectable effect of therapy (score 3). The treatment effect score is part of standard reporting in rectal carcinoma pathology and predicts outcomes, including local recurrence, metastasis, and recurrence-free survival (37, 49). To assess the possible relationship between commensal bacteria and treatment response, we correlated mucosal microbiome composition at the proximal margin with the treatment effect score, focusing the analysis only on subjects treated with neoadjuvant chemoradiation (*n* = 40, Fig. 4A). Clinicopathologic features by neoadjuvant response are described in Supplementary Table S2. α diversity did not correlate with the treatment scores using several metrics. However, β diversity (UniFrac distance) was significantly different between cases with no residual viable carcinoma (complete response, score 0) and cases with more viable carcinoma remaining after neoadjuvant therapy (PERMANOVA, *P* = 0.001). Complete and incomplete treatment response specimens clustered separately in the principal component analysis (Fig. 4B). Analysis of specific taxon abundances identified several taxonomic units that were differentially abundant in the specimens according to the treatment response score (Fig. 4C). Taxonomic units from the *Streptococcus* genus, *Lachnospiraceae*, and *Bacillaceae* were depleted in the mucosae of patients with a complete pathologic response (Fig. 4D–F), whereas *Pseudomonadota* were increased. We conclude that the composition of the mucosal microbiota differs substantially according to pathologic response after neoadjuvant chemoradiotherapy.

### Predicted bacterial metabolic pathway enrichment in complete responders

**Figure 5:**
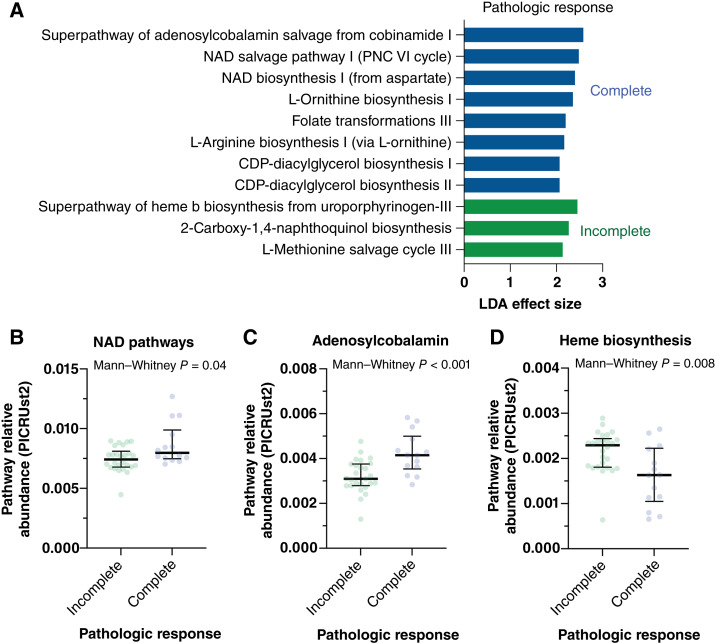
Predicted metabolic pathways associated with host radiation responses are enriched in complete pathologic responder microbiomes*.* Metabolic pathway gene quantities were predicted from 16S rDNA sequence data using PICRUSt2, and enrichment testing was done by LDA (**A**, LEfSe; refs. 42, 43). Predicted NAD salvage and biosynthesis (**B**), cobinamide-related metabolism (**C**), and L-arginine/ornithine biosynthesis pathways were enriched in complete responders, whereas bacterial heme b synthesis was most enriched in incomplete responders (**D**).

To assess the response of mucosal bacteria to chemoradiation in complete responders, we used a 16S taxonomy-based approach [PICRUSt2 (43)] to predict metabolic pathway enrichment (Fig. 5A–D). Bacterial NAD^+^ biosynthesis and salvage, an adenosylcobalamin salvage pathway, and arginine/ornithine metabolism pathways were enriched in communities from specimens with a complete response, whereas a bacterial heme synthesis–related pathway was enriched in incomplete responders. Host NAD^+^ is required for response to oxidative stress and PARP-mediated DNA repair in the setting of radiation damage, and NAD^+^-targeted therapies may enhance radiotherapy response (50). *Pseudomonadota* salvage cobinamide, a critical enzymatic cofactor for the synthesis of cobalamin. In the host, cobinamide is a vitamin B12 precursor that is also radioprotective via antioxidant mechanisms (51). Reduced circulating cobalamin levels have been observed in patients undergoing radiotherapy for rectal cancer (52). The production of L-ornithine from arginine modulates mucosal immunity and promotes mucus layer homeostasis, an effect reversed by mutation of biosynthetic enzymes in *Lactobacillus* (53). Enrichment of these bacterial pathways in irradiated specimens suggests adaptation of the mucosal community to radiation-related oxidative stress, which differs between complete and incomplete responders.

## Discussion

In summary, we found that the mucosa-associated microbiota at the proximal margins of rectal carcinoma resection specimens from patients with symptoms of radiation colitis differed from those of other neoadjuvant treatment groups. Several taxa, including the phylum level groups, were differentially abundant, with the most prominent magnitude of difference observed in the radiation-induced colitis group. Sequences derived from the *Bacillaceae* family were strikingly increased in patients who underwent radiotherapy and developed radiation-induced colitis. Most importantly, mucosa-associated microbiome diversity reflected the pathologic neoadjuvant treatment effect score. A complete response to neoadjuvant chemoradiation is marked by distinct mucosal community β diversity, including depletion of *Streptococcus*, *Lachnospiraceae*, and *Bacillaceae*.

Chemotherapeutic agents contribute to the symptoms of mucositis and have been associated with fecal microbiome alterations (54). For example, treatment with 5-FU, a cornerstone colorectal carcinoma therapy, significantly altered both α and β diversity in mice (55). Growth of the pathobiont *F*. *nucleatum* is inhibited by 5-FU, and tumor-associated bacteria, such as some *Escherichia coli* strains, modify 5-FU to reduce its tumoricidal activity (56). The other dominant chemotherapeutic agent used in our population, capecitabine, had no significant effect on commensal bacteria during the treatment of patients with colorectal carcinoma in separate studies (57). These prior studies highlight an important strength of the present study: control of oncologic treatment variables, including neoadjuvant chemotherapy-only and nontreated comparators. The chemotherapy-only group was younger and more likely to be treated with immunotherapy (Table 1), which could confound comparisons with the other groups. The smaller number of patients in the chemotherapy only and no neoadjuvant groups was sufficient to statistically power microbiome comparisons but may limit generalizability to other populations. Importantly, all characteristics in Table 1 were indistinguishable in the larger radiation with and without colitis groups, which are the primary comparisons for outcomes of colitis symptoms and response to neoadjuvant therapy.

The high relative abundance of *Bacillaceae* family members corresponded to two adverse outcomes in our cohort: radiation colitis and incomplete pathologic response. The enrichment of fecal *Bacillaceae* in radiation colitis has been observed in previous studies. A study of patients with locally advanced rectal carcinoma also found an enrichment of fecal *Bacteroides* and *Bacilli* after irradiation (15). Consistent with our findings, a prior prospective study of chemoradiation-related rectal swab microbiome changes in patients with gynecologic cancers identified increased *Bacilli* (58). Enrichment of *Anaerobacillus*, a member of *Bacillaceae*, has been described in pretreatment rectal carcinoma biopsies from patients with an incomplete response to subsequent neoadjuvant therapy (59). Possible mechanisms for commensal bacterial influence on the development of radiation colitis include modulation of immune responses, e.g., through Toll-like receptor activation (60), altered SCFA production (20), effects on epithelial barrier function (61), and opportunistic pathogenicity of specific taxa. Taken together, these findings suggest that patients with high relative mucosa-associated *Bacillaceae* before treatment are less likely to have a complete pathologic response, and the mucosal enrichment of this taxon is also apparent after treatment.

We observed a relative enrichment of *Lachnospiraceae* with an incomplete pathologic response to neoadjuvant therapy (Fig. 4C). Changes in the abundance of *Lachnospiraceae* after radiation have been described in several studies and model systems, increasing in some and decreasing in others (62). *Lachnospiraceae* and their SCFA metabolites have been causally implicated in the protection of the intestinal mucosa and other organ systems in a rodent total body irradiation model (27). Furthermore, in a rodent subcutaneous injection model of colorectal cancer, intestinal colonization with *Lachnospiraceae* or treatment with butyrate was sufficient to reduce the tumor response to radiation (63). Thus, *Lachnospiraceae* and their metabolites may confer epithelial protection against radiation injury, which is desirable in total body irradiation and environmental exposures (27). However, *Lachnospiraceae* may confer unwanted protection of rectal carcinoma cells in neoadjuvant chemoradiotherapy. Supporting this concept, a recent intratumoral microbiome study of young-onset colorectal cancer also found increased *Lachnospiraceae* in incomplete responders to neoadjuvant therapy (64). The mechanistic hypothesis remains to be tested and may have ramifications for using *Lachnospiraceae* probiotics in radiation colitis, in the context of rectal cancer therapy.

The predicted pathway enrichment in complete response versus incomplete response subjects (Fig. 5) raises several speculations. NAD^+^ is a ubiquitous host and cancer cell metabolite that plays a central role in mitigating oxidative stress and DNA repair in response to radiation (50). Adaptations of the neoplasm to increased NAD^+^ production have been linked to radiation resistance (65). One explanation for the enriched bacterial NAD salvage and biosynthesis in complete responders is adaptation to NAD^+^-poor, radiation-sensitive tumor environments. Altered bacterial pathways with substrates (cobinamide) and products (L-ornithine) involved in mitigating oxidative stress and mucosal reparations suggest adaptation of the mucosal microbiome to host tissue radiation responses, which may differ between the complete and incomplete pathologic response groups.

All specimens included in our study were obtained from posttreatment resections. A few studies have demonstrated that microbiome features preceding neoadjuvant therapy can aid in predicting treatment response and symptoms (15, 24). A recent study identified taxa such as *Streptococcus* and several *Lachnospiraceae* (*Blautia*, *Roseburia*, and *Dorea*) were enriched in pretreatment mucosal biopsies from patients with late-onset rectal carcinoma who did not achieve a complete pathologic response to neoadjuvant therapy (64). This microbiome profile aligns well with our posttreatment comparison by pathologic response (Fig. 4).

Our study highlights microbiome changes after neoadjuvant therapy that can help distinguish complete from incomplete pathologic responses. These features may add valuable information to guide the selection of patients for nonoperative management (“watch and wait”). Ultimately, a clinical microbiome-based test for this purpose would be performed on fecal or mucosal biopsy specimens. Further studies are needed to determine whether the β diversity distinction between complete and incomplete responder microbiomes (Fig. 4) holds for nonresection specimen types.

### Conclusions

We found that the mucosal microbiome diversity is altered in patients with rectal carcinoma following neoadjuvant therapy. Microbiome profiles correspond to symptoms of radiation colitis, histologic evidence of radiation injury to the intestine, and most importantly, the histologic treatment response score. Complete pathologic treatment response (no residual carcinoma) corresponds to robust mucosal microbiome β diversity and metabolic pathway differences, which may provide prognostically useful biomarkers. Further studies are needed to assess the causal relationships and mechanisms of mucosal dysbiosis and radiation colitis and whether these alterations can be targeted with microbiome-directed therapies.

## Supplementary Material

Table S1 Table S1

Table S2 Table S2

Figure S1 Figure S1
