# Supplementary material for: Mucosal Microbiome Markers of Complete Pathologic Response to Neoadjuvant Therapy in Rectal Carcinoma
Source: Cancer Res Commun. 2025 May 5;5(5):756–66. doi: 10.1158/2767-9764.CRC-25-0036 (PMC12051095; doi:10.1158/2767-9764.CRC-25-0036)
Supplement: Table S1 [file crc-25-0036_table_s1_suppst1.docx]

| **Taxon classification** | **Identification method** | **Read fraction** |
| --- | --- | --- |
| k_Bacteria, not further classifiable | frozen / FFPE comparison, decontam | 0.13 |
| g_Corynebacterium | frozen / FFPE comparison, decontam | 0.02 |
| f_Moraxellaceae | frozen / FFPE comparison, decontam | 0.02 |
| f_Xanthomonadaceae | frozen / FFPE comparison, decontam | 0.02 |
| f_Microbacteriaceae | frozen / FFPE comparison, decontam | 0.01 |
| f_Propionibacteriaceae | frozen / FFPE comparison, decontam | 0.01 |
| p_Cyanobacteria | frozen / FFPE comparison, decontam | 0.009 |
| f_Nocardiaceae | frozen / FFPE comparison, decontam | 0.008 |
| f_Mycobacteriaceae | frozen / FFPE comparison, decontam | 0.007 |
| f_[Weeksellaceae] | frozen / FFPE comparison, decontam | 0.006 |
| o_Neisseriales | frozen / FFPE comparison | 0.005 |
| f_Flavobacteriaceae | frozen / FFPE comparison, decontam | 0.003 |
| Unassigned | frozen / FFPE comparison, decontam | 0.001 |

**Table S1.** *Taxonomic groups excluded from analysis as likely FFPE contaminants*. Contaminants were identified as over-representation in 10 FFPE specimens with paired fresh frozen tissue. These 10 specimen pairs were not from subjects of this study. PCR and sequencing were performed simultaneously for FFPE and fresh frozen tissue. In addition, a statistical method of contaminant detection was applied as previously described [35]. The predominant contaminant of unclassifiable sequences likely represents non-specific amplification of host and formalin-damaged DNA. Removing sequences assigned to these taxa resulted in 25% of reads excluded.
