# Supplementary material for: Mucosal Microbiome Markers of Complete Pathologic Response to Neoadjuvant Therapy in Rectal Carcinoma
Source: Cancer Res Commun. 2025 May 5;5(5):756–66. doi: 10.1158/2767-9764.CRC-25-0036 (PMC12051095; doi:10.1158/2767-9764.CRC-25-0036)
Supplement: Table S2 [file crc-25-0036_table_s2_suppst2.docx]

|  | **Chemoradiation**  **complete response** | **Chemoradiation incomplete response** | **Test** | **p** |
| --- | --- | --- | --- | --- |
| **Total subjects *n*** | 14 | 26 | NA | NA |
| **Sex** |  |  | Chi square | 0.6 |
| Male *n* | 7 | 15 |  |  |
| Female *n* | 7 | 11 |  |  |
| **Age *median* *yr (IQR)*** | 63 (59-75) | 62 (55-64) | Mann-Whitney | 0.4 |
| **Pathologic stage group *n*** |  |  | Chi square | 0.002 |
| 1 | 14 | 7 |  |  |
| 2 | 0 | 11 |  |  |
| 3 | 0 | 8 |  |  |
| 4 | 0 | 0 |  |  |
| **Radiation therapy** |  |  |  |  |
| Interval to resection *median* *days (IQR)* | 75 (63-133) | 108(74-217) | Mann- Whitney | 0.2 |
| 50.4 Gy / 28 *n (%)* | 14 (100) | 26 (100) | NA | NA |
| **Radiation colitis *n*** |  |  | Chi square | 0.1 |
| yes | 6 | 18 |  |  |
| no | 8 | 8 |  |  |
| **Chemotherapy *n*** |  |  | Chi square | 0.7 |
| 5-FU-based | 14 | 25 |  |  |
| capecitabine | 10 | 22 |  |  |
| oxaliplatin | 10 | 22 |  |  |
| panitumumab | 0 | 0 |  |  |
| bevacizumab | 0 | 0 |  |  |
| **Microsatellite status *n*** |  |  | NA | NA |
| Instability (MSI) | 0 | 0 |  |  |
| Stable (MSS) | 14 | 26 |  |  |
| **Post-resection follow-up** |  |  |  |  |
| Follow-up interval *median months (IQR)* | 20 (12-25) | 89 (71-107) | Mann-Whitney | 0.01 |
| Recurrence *n* | 0 | 2 | Chi square | 1 |
| Distant metastasis *n* | 2 | 1 | Chi square | 0.8 |

**Table S2.** *Study group demographics, stage, and oncologic treatment exposures by pathologic response.* Abbreviations: IQR interquartile range, Gy Gray, 5-FU 5-fluorouracil, MSS microsatellite stable, MSI microsatellite instability, yr years.
