# Supplementary figures and images for: Mucosal Microbiome Markers of Complete Pathologic Response to Neoadjuvant Therapy in Rectal Carcinoma
Source: Cancer Res Commun. 2025 May 5;5(5):756–66. doi: 10.1158/2767-9764.CRC-25-0036 (PMC12051095; doi:10.1158/2767-9764.CRC-25-0036)

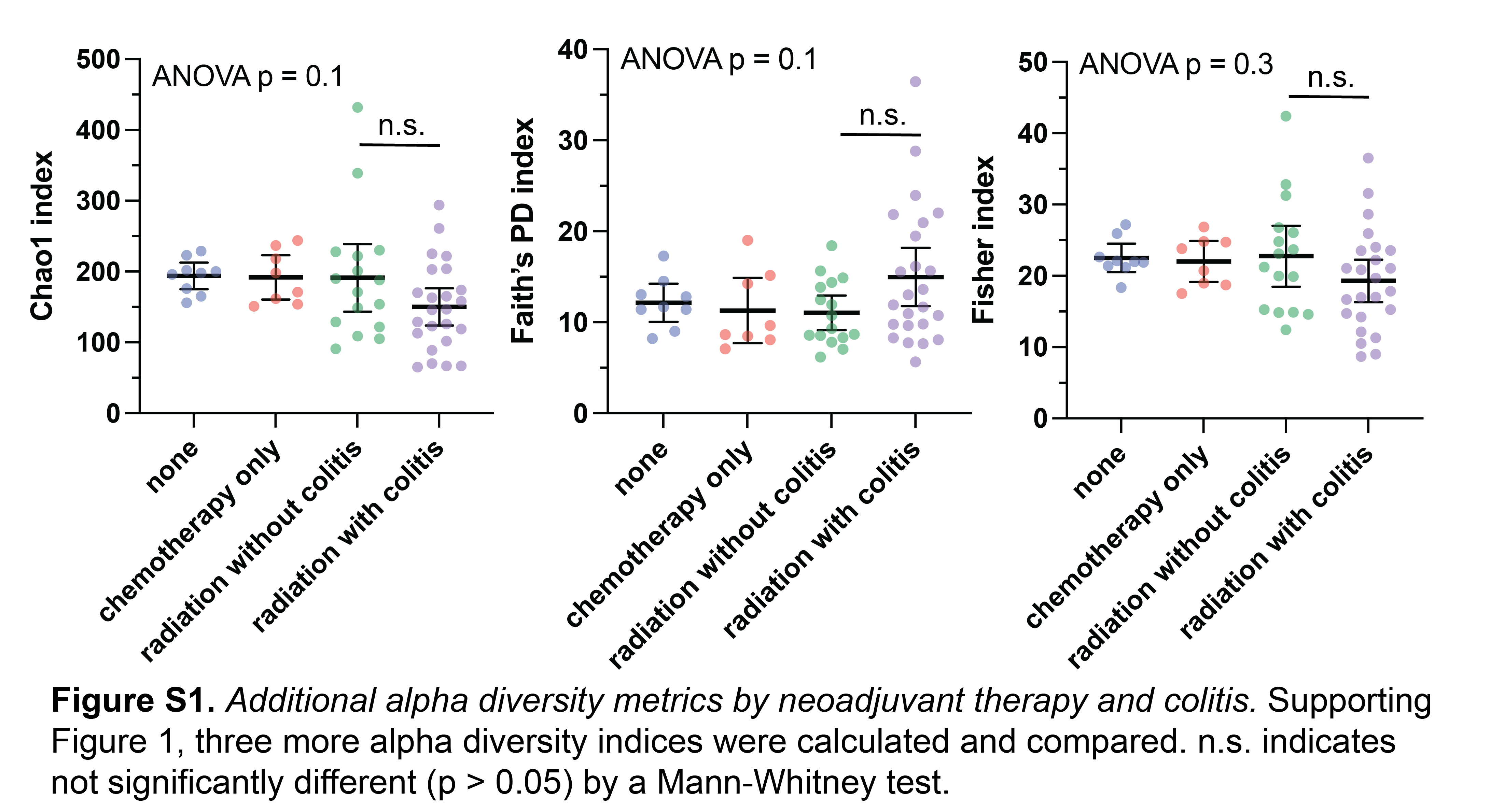

Supplement: Figure S1 [file crc-25-0036_figure_s1_suppsf1.png]
